# Electrocardiographic Screening for Prolonged QT Interval to Reduce Sudden Cardiac Death in Psychiatric Patients: A Cost-Effectiveness Analysis

**DOI:** 10.1371/journal.pone.0127213

**Published:** 2015-06-12

**Authors:** Antoine Poncet, Baris Gencer, Marc Blondon, Marianne Gex-Fabry, Christophe Combescure, Dipen Shah, Peter J. Schwartz, Marie Besson, François R. Girardin

**Affiliations:** 1 Department of Health and Community Medicine, University Hospitals and University of Geneva, rue Gabrielle-Perret-Gentil 4, 1205, Geneva, Switzerland; 2 Cardiology Division, University Hospitals and University of Geneva, rue Gabrielle-Perret-Gentil 4, 1205, Geneva, Switzerland; 3 Department of Internal Medicine, University Hospitals and University of Geneva, rue Gabrielle-Perret-Gentil 4, 1205, Geneva, Switzerland; 4 Department of Psychiatry, University Hospitals and University of Geneva, 2 chemin du Petit-Bel-Air, 1225, Chêne-Bourg, Switzerland; 5 Center for Cardiac Arrhythmias of Genetic Origin, IRCCS Istituto Auxologico Italiano, Milano, Italy; 6 Department of Anesthesiology, Intensive Care, and Clinical Pharmacology, University Hospitals and University of Geneva, rue Gabrielle-Perret-Gentil 4, 1205, Geneva, Switzerland; 7 Medical Directorate, University Hospitals and University of Geneva, rue Gabrielle-Perret-Gentil 4, 1205, Geneva, Switzerland; 8 Centre for Health Economics, University of York, Heslington,York, United Kingdom; University of Miami School of Medicine, UNITED STATES

## Abstract

**Importance:**

Sudden cardiac death is a leading cause of mortality in psychiatric patients. Long QT (LQT) is common in this population and predisposes to Torsades-de-Pointes (TdP) and subsequent mortality.

**Objective:**

To estimate the cost-effectiveness of electrocardiographic screening to detect LQT in psychiatric inpatients.

**Design, Setting, and Participants:**

We built a decision analytic model based on a decision tree to evaluate the cost-effectiveness and utility of LQT screening from a health care perspective. LQT proportion parameters were derived from an in-hospital cross-sectional study. We performed experts' elicitation to estimate the risk of TdP, given extent of QT prolongation. A TdP reduction of 65% after LQT detection was based on positive drug dechallenge rate and through adequate treatment and electrolyte adjustments. The base-case model uncertainty was assessed with one-way and probabilistic sensitivity analyses. Finally, the TdP related mortality and TdP avoidance parameters were varied in a two-way sensitivity analysis to assess their effect on the Incremental Cost-Effectiveness Ratio (ICER).

**Main Outcomes and Measures:**

Costs, Quality Ajusted Life Year (QALY), ICER, and probability of cost effectiveness thresholds ($ 10 000, $25 000, and $50 000 per QALY).

**Results:**

In the base-case scenario, the numbers of patients needed to screen were 1128 and 2817 to avoid one TdP and one death, respectively. The ICER of systematic ECG screening was $8644 (95%CI, 3144-82 498) per QALY. The probability of cost-effectiveness was 96% at a willingness-to-pay of $50 000 for one QALY. In sensitivity analyses, results were sensitive to the case-fatality of TdP episodes and to the TdP reduction following the diagnosis of LQT.

**Conclusion and Relevance:**

In psychiatric hospitals, performing systematic ECG screening at admission help reduce the number of sudden cardiac deaths in a cost-effective fashion.

## Introduction

Sudden cardiac death (SCD) due to ventricular arrhythmias is a leading cause of premature mortality in patients with psychotropic medications.[1] It is estimated that 2 to 3% of prescriptions may cause LQT and, possibly, ventricular tachyarrhythmias, such as *Torsades de Pointes* (TdP).[2] We have previously shown that drug-induced LQT is common among psychiatric inpatients, especially in those treated with antipsychotics, antidepressants, methadone, or taking illicit drugs.[3, 4] TdP is a transient but recurrent malignant polymorphic ventricular arrhythmias often deteriorating into ventricular fibrillation: each episode has been associated with a mortality of approximately 15% in psychiatric population and in patients with heart failure.[3, 5] As LQT is the main predisposing factor for TdP, its detection with the electrocardiogram (ECG) could identify individuals at substantial risk of developing TdP.[6]

Despite calls for physicians’ awareness of this problem [7] and for assessing risk by means of a QT normogram,[8] no consensual recommendation is available about the utility of screening drug-induced LQT with a resting 12-lead ECG in psychiatric populations. Given its low cost, wide use, and safety, we hypothesized that ECG screening may identify individuals with LQT in a cost-beneficial fashion. The effectiveness of ECG screening has already been demonstrated for the detection of atrial fibrillation, the reclassification of cardiovascular risk in the general population, and the identification of infants with long QT syndrome.[9–11] The latter screening, performed prospectively in >44 000 infants, was also demonstrated to be cost-effective leading to recommendations for widespread ECG screening in neonates.[12, 13]

To provide guidance on whether or not routine ECG screening might be recommended in patients hospitalized in psychiatric wards, we performed an economic evaluation to assess the cost-effectiveness of systematic LQT detection by ECG screening.

## Methods

### Study population and model structure

The study population was derived from 6790 men and women adults (mean age: 41 years) admitted in the public Psychiatric Hospital of Geneva, based on the ESOP study [3] –(Department of Mental Health and Psychiatry, University Hospitals of Geneva, Switzerland). Characteristics of patients with drug-induced long QT at admission to the psychiatric hospital and a subgroup with subsequent *torsade de pointes* or sudden death are detailed in Table 1. We built a decision analytic model to evaluate the cost-effectiveness of LQT screening in adult patients admitted to a psychiatric hospital. Because the incidence of TdP resulting from LQT is low, a prospective comparative study would need several thousands of patients: we used a *de novo* decision analytic model with 10 000 cohort simulations, each of them having 100 000 patients; we extrapolated the results over entire life.[3, 14]

**Table 1:** Characteristics of patients in the ESOP Study [3].

	Normal ECG (N = 143)		Drug-induced long QT (QTc ≥500 ms; N = 62)		Torsade de pointes (N = 7) or sudden death (N = 5)	
	median	range	median	range	median	range
Age	41	18–66	40	20–74	42	20–69
	n	%	n	%	n	%
Male sex	74	51.7	26	41.9	9	75.0
Obesity (body mass index ≥ 30, N = 140 and 61)	23	16.4	16	26.2	1	8.3
Smoker	77	69.4	32	60.4	4	40.0
Coronary heart disease	7	4.9	2	3.2	1	8.3
Hypokalemia (potassium < 3.5 mEq/L)	8	5.6	12	19.4	3	25.0
Hepatitis C virus infection	14	9.8	26	41.9	6	50.0
Human immunodeficiency virus infection	9	6.3	15	24.2	5	41.7
ICD-10 psychiatric diagnoses						
Disorders due to psychoactive substance use	42	29.4	29	46.8	7	58.3
Schizophrenia, schizotypal and delusional dis.	28	19.6	19	30.6	3	25.0
Mood disorders	59	41.3	19	30.6	2	16.7
Psychotropic medication ^a^						
Antipsychotics (any)	101	70.6	53	85.5	11	91.7
aripiprazole	7	4.9	3	4.8	0	0.0
clotiapine	6	4.2	9	14.5	3	25.0
clozapine	21	14.7	7	11.3	4	33.3
haloperidol	6	4.2	10	16.1	3	25.0
olanzapine	17	11.9	10	16.1	4	33.3
promazine / levomepromazine	12	8.4	19	30.6	2	16.7
quetiapine	33	23.1	10	16.1	0	0.0
risperidone / paliperidone	19	13.3	14	22.6	0	0.0
sertindole	2	1.4	7	11.3	1	8.3
Antidepressants (any)	59	41.3	30	48.4	8	66.7
citalopram / escitalopram	15	10.5	15	24.2	3	25.0
fluoxetine	4	2.8	9	14.5	1	8.3
trazodone	12	8.4	1	1.6	0	0.0
venlafaxine	13	9.1	3	4.8	1	8.3
Mood stabilizers (any)	35	24.5	5	8.1	1	8.3
Sedative drugs and tranquilizers (any)	86	60.1	32	51.6	5	41.7
Methadone	12	8.4	23	37.1	7	58.3

^a^ Only the most frequent antipsychotic and antidepressant drugs are detailed.

**Fig 1:**
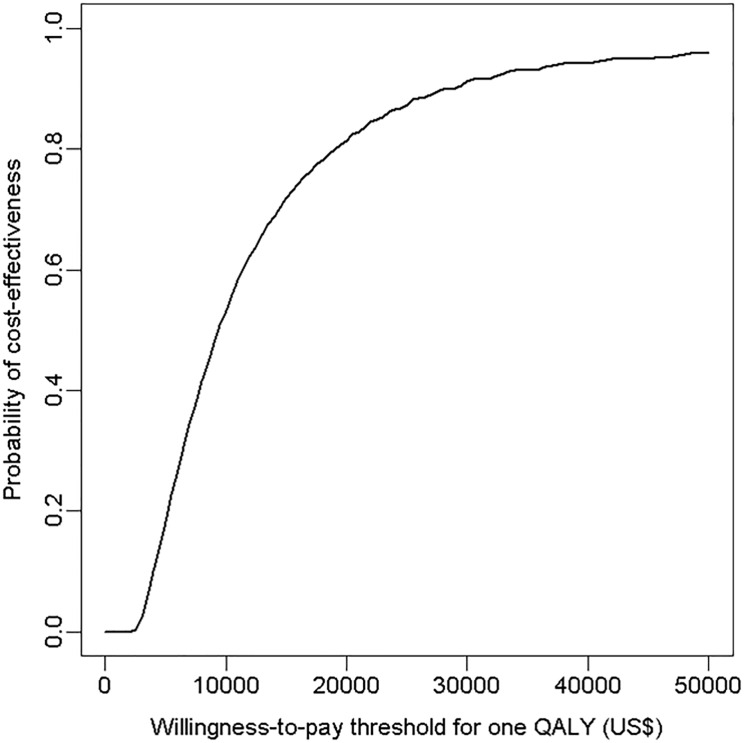
The decision tree represents both strategies: “ECG screening” at hospital admission *versus* “No ECG screening”. Probabilities of patients belonging to a QT category are identical in both strategies, as the risk of SCD after a TdP event. The probability of developing TdP is based on the severity of QT prolongation and is reduced by LQT detection in the ECG strategy. For patients remaining alive, the model assumes a life expectancy of 25 years.

The analytic model was a decision tree comparing two strategies (Fig 1): a “screening strategy” using systematic electrocardiographic LQT detection at hospital admission *versus* an “abstention strategy” (no routine ECG screening). In the “abstention strategy”, no screening was performed to identify patients with LQT. Patients with an unidentified LQT were at risk of arrhythmias and TdP. The pro-arrhythmic propensity depended on the length of QT. In the “screening strategy”, patients with LQT were identified by ECG and adequately managed (eg withdrawal of pro-arrhythmic drugs, supplementation of electrolytes, or magnesium aspartate).

We made the following assumptions: (1) patients without LQT had no risk of TdP; (2) only SCD related to TdP were accounted for specific LQT interval; (3) the time-frame of the model was life-long, but we assumed that for any patients with LQT, TdP episodes, and SCD would occur within one-year.

Modeled outcomes comprised one-year mortality, mean costs per patient, mean quality-adjusted life-years (QALYs) per patient, representing the quality-adjusted life expectancy of the target population and cost-effectiveness incremental ratios (ICER), in agreement with the current recommendation.[15] While the decision threshold value for one additional QALY is somewhat country-dependent, ICERs provide evidence for implementing guidance which maximizes health benefit, given available resources.[16] The model was developed using the R programming software (R Foundation for statistical computing, Vienna, Austria, Version 2.15.2).

### Probabilities

We estimated baseline probabilities for the base-case analysis, ranges for one- and two-way sensitivity analyses, and distributions for probabilistic analyses. The base-case analysis was conducted using baseline probabilities and costs, resulting in base-case results of difference of mortality, incremental effectiveness, incremental costs, and ICER.

#### Prevalence of LQT

The prevalence of LQT was determined from the ESOP study.[3] Patients were categorized into three pro-arrhythmic subgroups based on the extent of corrected QT prolongation (LQTc): mild LQTc (3.7%, range 480–520ms), intermediate LQTc (0.6%, range 520–600ms), and severe LQTc (0.1%, >600ms).

#### Risk of Torsades-de-Pointes

As the risk of TdP in patients with LQT is poorly characterized in the literature, it was derived from an experts’ elicitation based on a previously described methodology.[17] In January 2014, we approached 13 experts from the Universities of Geneva and Lausanne, Switzerland, and Milano, Italy, including 11 cardiologists (of whom 7 were electrophysiologists), and 2 psychopharmacologists. Each expert was individually and independently asked to estimate the risk of TdP for patients with the three categories of LQT within a one-year period after the occurrence of LQT. We used the histogram method by presenting plausible ranges of values partitioned into intervals of 1%, and asking experts to estimate the probability of TdP risk for each interval. Experts’ opinions were then combined by taking the arithmetic mean of individual assessments (linear pooling with equal weighting of expert), resulting in the following baseline mean risk of TdP: 2.3% (patients with mild LQTc), 5.7%, (patients with intermediate LQTc), and 13.6% (patients with severe LQTc). Our questionnaire and elicitation results are available in S1 Table and S1 Fig.

#### Influence of LQT detection on risk of TdP

Based on in-site psychopharmacological and pharmacovigilance follow-up, the QT duration normalized in 65% of patients with identified LQT (drug positive dechallenge). When the LQT was not adequately managed (35%), we assumed that the risk of TdP and SCD were similar to the “abstention” strategy. Therefore, we estimated that patients with detected LQT would have a 65% lower risk of TdP than in patients with undetected LQT. Since there is a large ECG interpretation heterogeneity across physicians and hospital settings, we chose a wide range between 5 and 100% in one- and two-way sensitivity analyses to highlight the uncertainty around this estimate.

#### Risk of sudden cardiac death

Findings from previous studies indicated an average mortality of 15% per episode and 3.4 episodes on average per patient experiencing TdP.[3, 5] SCD might result from ventricular fibrillation after the first TdP episode or, more likely, after recurrent TdP episodes. Although most TdP episodes revert spontaneously,[18] aggregated TdP related mortality per patient set at 40% (ie, [1-(1–0.15)^3.4^] = 0.4). We chose a large range from 2 to 60% in one- and two-way sensitivity analyses to capture the uncertainty surrounding this parameter.

### Costs

Taking a health-care perspective, our model simulated direct medical expenditures: costs of a onetime ECG screening, estimated at $35 from the Swiss reimbursement tariff catalog (Tarmed, 2014)[19] and consistent with other national schedules, such as the US Medical Fee Schedules ($33). We integrated the costs of additional medical attention related to the presence of LQT and to the medical management of TdP episodes: costs of our standard LQT management within the psychiatric ward by a specialist, including a 20-minute consultation by a psychopharmacologist or cardiologist ($127) and two follow-up ECGs ($70).[19] The total cost for management of a LQT was $197, rounded to $200.

Because TdP are potentially fatal, we assumed that all identified events required an intensive care unit admission for close rhythm monitoring until QT normalization: the aggregated costs of TdP management were estimated at $7700, derived from Hospital Episode Statistics and Diagnostic Related Group cost weights based on the SwissDRG webgrouper.[20] However, some TdP episodes could remain untreated and we empirically estimated that 70% of arrhythmias would be managed in the intensive care unit (unpublished inhospital data).

All cost estimates were given ranges for sensitivity analyses based on published variability of estimates. Self-limited and silent TdP generated no extra costs. The adaptation of psychotropic treatments to reduce the QTc prolongation was considered marginal. All cost results are reported in US$, with a rounded exchange rate of US$1 = 1 Swiss franc (January 15, 2015). Discount rates of 3% were applied to QALYs and costs.[21]

### Life expectancy and Quality-of-life

QALY was estimated by the time spent alive weighted by the utility value attributed to psychiatric conditions. Based on the mean age of 41 years of patients admitted to psychiatric wards,[3] and an overall mean life expectancy of 66 years, we assumed a life expectancy after ECG screening of 25 years in patients without SCD.[14] The utility, representing the quality-of-life with a value from 0 (death) to 1 (perfect health), was derived from index scores for chronic conditions (Table 2).[22]

**Table 2:** Input parameters—Base case values and ranges.

Parameters	Base case parameters	Range for sensitivity analysis^╪^	Probabilistic sensitivity analysis and modeling^†^	Source
**Prevalence of long QT**	**4.4%**		Yes: Beta distribution	[3]
QT in 480–520ms (%)	**3.7%**		Yes: Beta distribution	[3]
QT in 520–600ms (%)	**0.6%**		Yes: Beta distribution	[3]
QT >600ms (%)	**0.1%**		Yes: Beta distribution	[3]
**QT dependent risk of TdP per patient**				
Corrected QT [480–520ms]	**2.3%**		Yes: expert opinions’ combined distribution	elicitation
Corrected QT [520–600ms]	**5.7%**		Yes: expert opinions’ combined distribution	elicitation
Corrected QT [≥ 600ms]	**13.6%**		Yes: expert opinions’ combined distribution	elicitation
TdP rate reduction with ECG	**65%**	5–100	No	expert opinion
Sudden Cardiac Death	**40%**	2–60	No	[3]
**Utility** **§**				
Quality of Life (QoL)	**0.73**	0.51–0.95	No	[22]
**Costs**				
ECG (per record)	**$35**	20–50	No	[19]
LQT management (per patient)	**$200**	100–300	No	[20]
TdP management (per patient)	**$7 700**	5900–9500	No	[20]
TdP management	**70%**	50–88	No	inhospital data
Discount rate (per year)	**3%**	0–5	No	[21]
Life expectancy of patients without SCD	**25 y**	10–40	No	[14]

All costs are in US$.

^╪^ Range of values for which the parameters were varied in the one-way sensibility analysis.

^†^This column indicates whether or not parameter values were drawn at random from a distribution probability (S1 Table).

^§^Data for utilities represent the quality-of-life estimates for aggregated health states in psychiatry and range from 0 (death) to 1 (perfect health).

#### One-and two-way sensitivity analyses

We performed one-way sensitivity analyses to challenge the robustness of the results by varying the following parameters of the model throughout their credible ranges: TdP mortality and proportions of TdP avoided; quality-of-life; costs of ECG, LQT, TdP, and ambulatory management; discount rate; life expectancy of patients without SCD (Table 2). Considering the uncertainty around the TdP related mortality and TdP avoidance after LQT detection, we explored simultaneously the effect of both parameters on ICER in a two-way sensitivity analysis by jointly varying them over a large set of values.

#### Probabilistic sensitivity analyses

Probabilistic sensitivity analyses were conducted to explore joint parameter uncertainty of the model and whether parameter variability was translated into outcome variability. We assigned beta distributions to the proportion of LQT categories (S1 Table). The distribution for the pro-arrhythmic risk in each category of LQT was obtained by pooling distribution reported by the affiliated experts. For each of 10 000 simulated cohorts, values of parameters were drawn at random from the assigned distributions. The 95% CIs around the estimated mean costs per patient, ICER, QALY, and other effectiveness statistics were obtained by the percentiles 0.025 and 0.975 of 10 000 simulated cohorts. Results are presented by Cost Effectiveness Acceptability Curves (CEAC), which display the probability for the “screening” strategy to be cost-effective throughout a range of given willingness-to-pay (WTP) thresholds, and by a cost-effectiveness plane, which display the distribution of simulations on a scatterplot (incremental effectiveness (x-axis) and incremental costs (y-axis), S2 Fig).

### Role of the Ethics Committee and Source of Funding

The ESOP study protocol was approved in March, 2010, by the Ethics Committee of the Department of Mental Health and Psychiatry (protocol number Psy 09–025R) and the General College of Physicians, University Hospitals of Geneva. The protocol conformed to the 2008 Declaration of Helsinki. Written informed consent was not given by participants (because of the retrospective nature of pharmacovigilance) for systematic recording, but patient records/information was anonymized and de-identified prior to analysis. The funder of the study had no role in study design, data collection, data analysis, data interpretation, or writing of the report. The corresponding author had full access to all data in the study and final responsibility for the decision to submit for publication.

## Results

### Base case scenario

On average, in a cohort of 100 000 patients admitted to psychiatry wards, 4 400 had a LQT. In the “abstention strategy”, 136 developed TdP episodes, resulting in 54.6 SCD. In the “screening strategy”, 48 patients developed TdP spells, resulting in 19.1 SCD. This translated in numbers needed to screen of 1 128 patients to avoid one TdP, and 2 817 patients to avoid one SCD (Table 3). The average per patient costs related to the management of LQT was $8.9 in the “screening” strategy, exceeding the average per patient costs related to the management of TdP in the “abstention” strategy ($6.3), meaning the screening strategy generates more costs than it can save, even if ECG were free of charge. Adding the costs of ECG screening resulted in an incremental cost of $39.8 for the screening strategy. Screening yielded greater effectiveness, with an incremental 0.005 QALY (two quality-adjusted days), than no screening. The final ICER of the base-case analysis was $8 644 per incremental QALY.

**Table 3:** Base-case scenario results: Data in brackets are 95% CIs obtained from probabilities analyses.

Outcomes	« LQT screening » strategy	« No screening » strategy
Number of TdP per 100 000	48 [6; 114]	136 [19; 320]
Number of SCD per 100 000	19.1 [2; 47]	54.6 [7; 130]
Cumulated mortality (%)	0.019 [0; 0.047]	0.055 [0.007; 0.130]
NNT to avoid one TdP*	1 128	-
NNT to avoid one SCD*	2 817	-
Total cost per patient	$46.1 [43.8; 49.5]	6.3 [0.8; 14.8]
ECG Cost per patient	$35	0
Long QT Cost per patient	$8.9 [7.9; 9.9]	0
TdP Cost per patient	$2.2 [0.3; 5.2]	6.3 [0.8; 14.8]
QALY per patient	12.968 [12.964; 12.970]	12.963 [12.953; 12.969]
ICER (US$ per QALY) *	8 644 [3.144; 82.498]	-

*No screening is the reference strategy.

### One- and two-way sensitivity analyses

**Fig 2:**
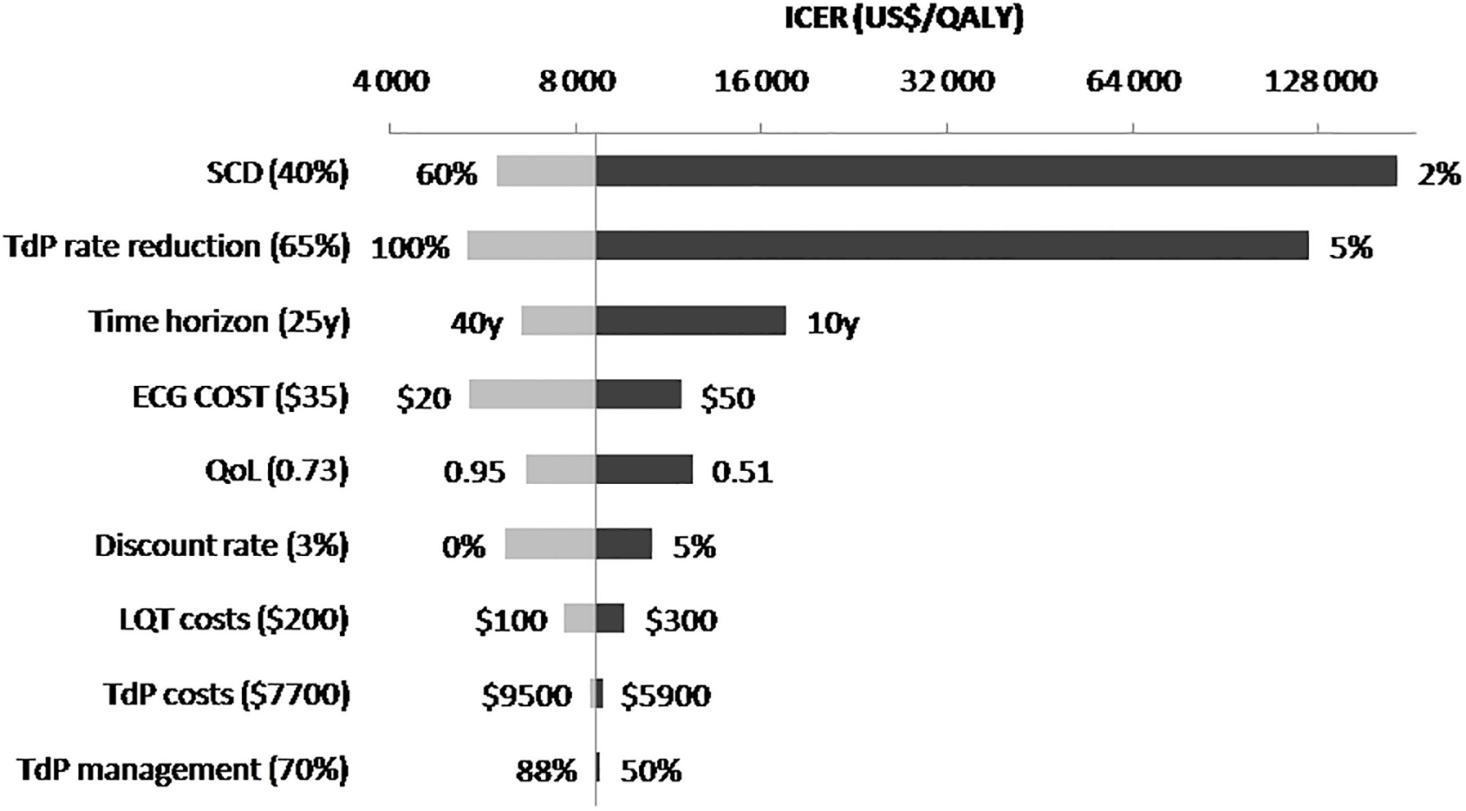
The tornado diagram represents the impact on the ICER when varying one single parameter (one-way sensitivity analysis). The vertical line represents the ICER in the base-case analysis (8644 US$/QALY) and the horizontal bars represent the variation of the ICER given variations of key parameters driving the model outcomes. The ranges of variations are represented by means of lower (light grey bars) and higher (dark grey bars) ICER values, with the base-case scenario parameter values in the midline indicated in brackets.

**Fig 3:**
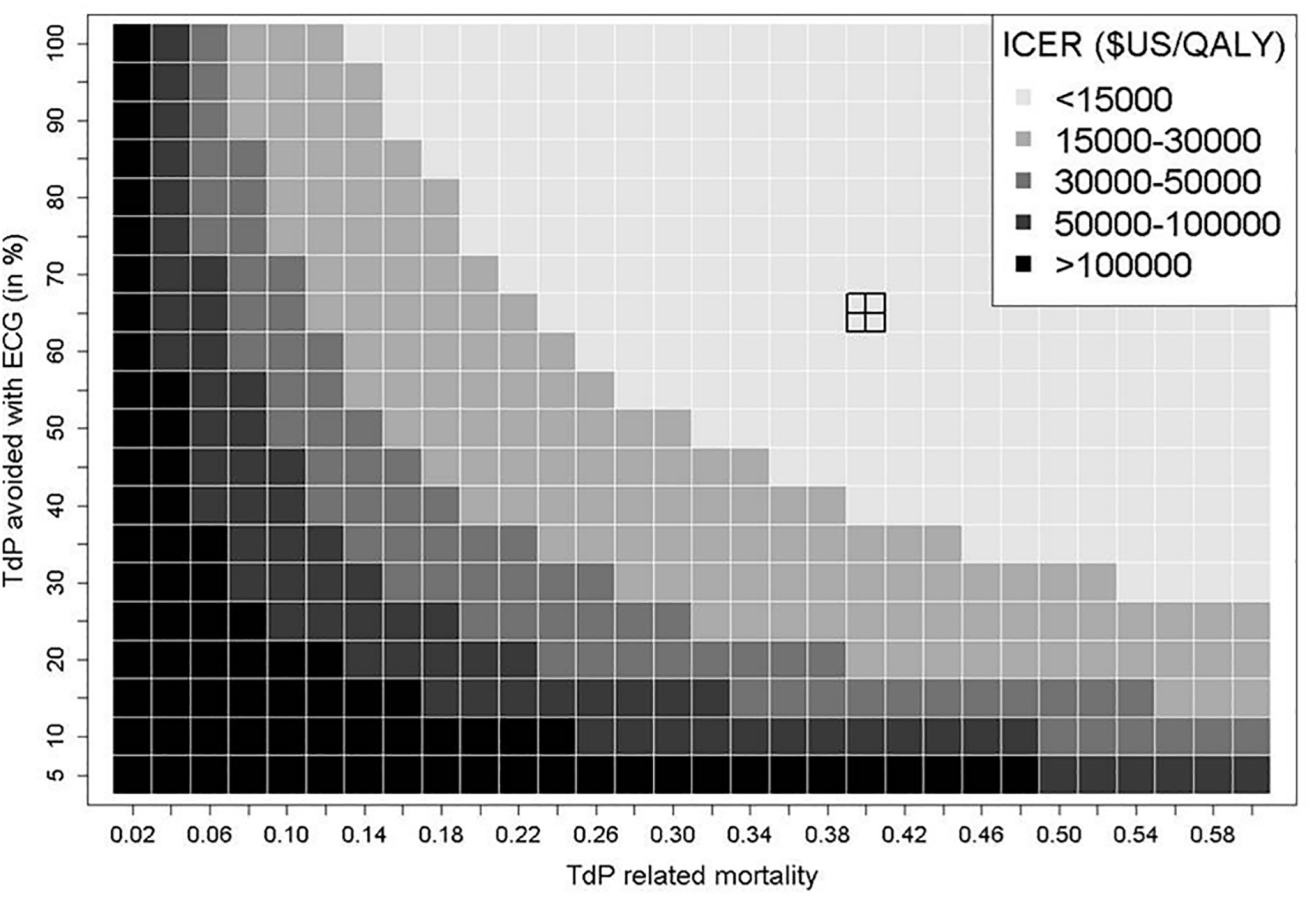
The two-way sensitivity analysis displays the impact on ICER of jointly varying the risk reduction of TdP after LQT detection and the TdP mortality. The highlighted target square represents the resulting ICER ($8644/QALY) from the base case scenario. The shaded colors represent the magnitude of the ICER from grey representing the lowest ICERs (<$15000/QALY) to darkest shades representing highest ICERs (>$100000/QALY).

In one-way sensitivity analyses, we checked the robustness of the model by varying parameters throughout their plausible ranges (Fig 2). The results were not sensitive to variations in life expectancy, utility values, and costs of ECG, LQT, and TdP management. However, we observed that the ICER was sensitive to variations in estimates of TdP mortality and the TdP risk reduction after detection. Fig 3 presents a two-way sensitivity analysis of the latter parameters. The most extreme scenarios yielded dramatically different ICERs, from $3 749 per gained QALY (TdP related patient mortality of 60% and 100% risk reduction of TdP with screening) to $2 787 101 per gained QALY (TdP related patient mortality of 2% and 5% risk reduction of TdP with screening). The ICER of the ECG strategy was always under $15 000 per QALY if one assumes a TdP related mortality >20%, jointly with a TdP rate reduction >75%.

### Probabilistic analysis

**Fig 4:**
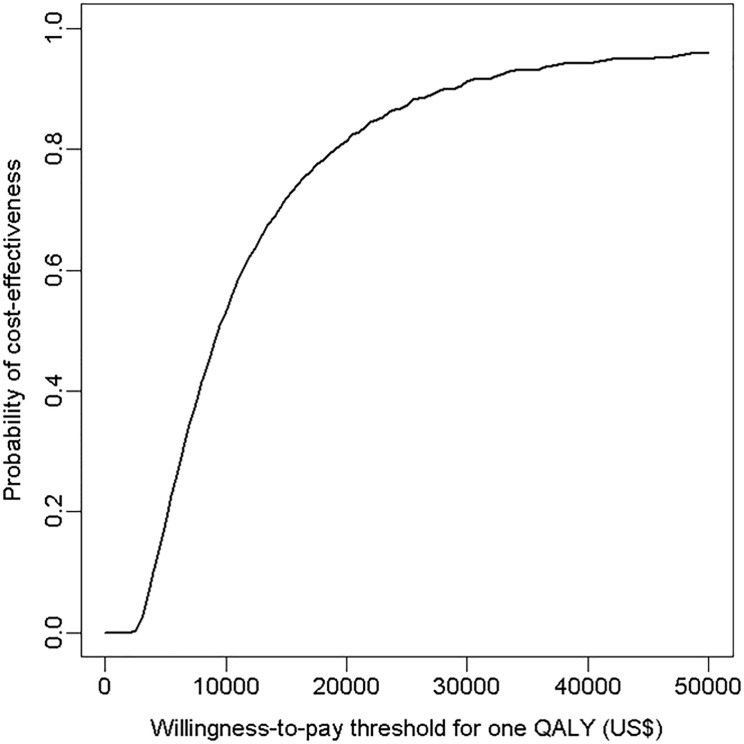
Cost Effectiveness Acceptability Curve (CEAC). This curve represents the probability of the long QT strategy of being cost effective under various ranges of willingness-to-pay threshold values per QALY. The strategy “ECG” in the base case scenario is cost effective in 86% and 96% of the simulated cohorts at willingness-to-pay thresholds of $25 000 and $50 000, respectively.

Simulations from 10 000 cohorts of 100 000 patients yielded incremental costs and incremental effectiveness estimates of $39.8 (95% CI 34.2 to 43.5) and 1.68 (95% CI 0.19 to 4.02) quality-adjusted days, respectively. The ICER was $8 644/QALY (95% CI 3 144 to 82 498) (Table 3). In the cost-effectiveness plane, all simulations were located in the northeast quadrant of the cost-effectiveness plane (more effective but more costly) (S2 Fig). In Cost-Effectiveness Acceptability Curves (Fig 4), the probability that the “screening” strategy was cost-effective was 51%, 86% and 96% for a willingness-to-pay of $10 000, $25 000, and $50 000 per QALY, respectively.

## Discussion

The present study has estimated possible benefits and cost-effectiveness ratios of a widely available intervention, an ECG screening in adult psychiatric patients to prevent mortality related to LQT. The results suggest that one death may be prevented by screening 3 000 patients at admission and thus identifying and treating patients pro-arrhythmic propensity. Despite the relatively high number of patients needed to screen, the LQT detection appears cost-effective, with an ICER of $8 644 per QALY, ie a substantially lower value than usual decision thresholds per QALY gained for health-care interventions in Europe or the United States ($ 50 000-$100 000 per QALY gained).[12, 23] This is explained by the inexpensiveness of the ECG screening. Interestingly, this ICER value is similar to that observed for neonatal ECG screening.[12]

The European Society of Cardiology (ESC) guidelines recommend 12-lead ECG follow-up in patients receiving anti-arrhythmic drugs,[24] but no specific recommendations are given for patients treated with psychotropic medications. This is of particular concern since antipsychotics and other psychotropic drugs clearly increase the risk of SCD.[25–27] In a hospitalized psychiatric population, we have previously shown that LQT was not uncommon and associated with conditions such as hypokaliemia, hepatitis C virus infection, HIV infection, ECG abnormalities (abnormal T wave morphology), and drugs such as clotiapine, haloperidol, promazine/levomepromazine, sertindole, citalopram/escitalopram, fluoxetine and methadone. The cost-effectiveness of ECG in the psychiatric hospital might depend on such population characteristics, as well as concomitant cardiovascular disease and liver or kidney dysfunction that may interfere with drug elimination.

The corrected QT interval according to Bazett’s formula is a well-known surrogate marker used to assess the risk of developing TdP.[8] It is probable that events related to LQT are underreported in clinical practice and active surveillance would increased awareness and better sensitize physicians to declare those life-threatening drug-induced adverse events by means of updated web access. In addition to clinical indications, QT interval prolongation also has a major impact on the drug regulation and the pharmaceutical market and is still one leading cause for drug withdrawal. However, more data are needed to better understand the profile of patients at higher risk of developing LQT, TdP episodes, and SCD. The integration of genetic and pharmacogenomic tests could offer better identification of patients at risk of LQT and subsequent ventricular arrhythmia. Recent genome-wide association studies suggest promising insights into the understanding and predisposition of drug-induced LQT: whole exome sequencing analysis implicates an increased burden of multiple rare potassium channel variants in the risk of developing fatal TdP.[28, 29] It remains to be determined whether the search for genetic polymorphisms or rare variants might be cost-effective in some high-risk individuals and, possibly, generalized to larger scale.

Our decision-analytical model has strengths and limitations. Strengths of this analysis to assess the cost-effectiveness of systematic ECG include the data sources for the prevalence of LQT categories derived from a five-year cross-sectional study to detect drug-induced LQT in a large population of adult psychiatric inpatients. In addition, the results of the model were robust over a wide range of plausible scenarios of TdP mortality and proportions of TdP avoided.

Decision analytic models are inherently limited by the uncertainty of their parameters; we found that our results were sensitive to two clinical parameters: (1) the case-fatality of TdP and (2) the risk reduction of TdP following ECG. The first parameter depends on the risk of TdP according to the duration of LQT as well as the mortality following one single episode of TdP, both of which remain poorly characterized. We chose to derive these probabilities from experts’ elicitation and from previous study results.[3] Thus, a cumulative TdP case-fatality of 40% was estimated and, based on experts’ elicited risks of TdP for a given LQTc, our model therefore suggested an incidence of 55/100 000 patient-years without ECG screening. Ray conducted a large retrospective cohort study of Medicaid antipsychotic drug users and observed an incidence of SCD of 179/100 000 patient-years.[25] Although the study population and design are not comparable, this incidence suggests that a rather conservative base-case scenario was used for our analysis. This adds strength and reliability to our findings.

The second key parameter was the estimated risk reduction of TdP after ECG recording. We hypothesized that the ECG based detection of LQT by clinicians would result in clinical measures to correct ECG abnormality. For instance, the clinician can switch pro-arrhythmic drugs to a safer medication or, alternatively, reduce the dosage of LQT promoting drug. [3, 25] Correction of other medical conditions, such as electrolytic disorders, treating chronic infection (ie Human Immunodeficiency and Hepatitis C virus infections), could decrease the arrhythmias proclivity. Even with the automatic interpretation of ECG records, the validity of ECG findings might be lower in clinical practice, especially if patients’ characteristics are different from our study population. Further, the measurement of LQT might present some inter-observer variability across physicians. Therefore, we chose an estimate of 65% for TdP reduction rate after LQT detection as base-case scenario.

In conclusion, we found that ECG screenings bring safety benefits with limited resource consumption and that systematic ECG to detect LQT is a cost-effective strategy in psychiatric wards for the prevention of sudden cardiac death. This is particularly relevant in the context polymedication with a wide range of psychotropic drugs and other illicit compounds. These findings could provide guidance to lessen premature mortality of psychiatric patients.

## Supporting Information

S1 Fig Representation of the linear pooling distribution of 13 experts for the mild, intermediate, and severe LQT categories. (TIF)

S2 Fig Cost-effectiveness plane of the probabilistic sensitivity analysis. (TIF)

S1 Table Supplemental Information. (PDF)
