# Correction: Electrocardiographic Screening for Prolonged QT Interval to Reduce Sudden Cardiac Death in Psychiatric Patients: A Cost-Effectiveness Analysis

**DOI:** 10.1371/journal.pone.0133108

**Published:** 2015-07-14

**Authors:** Antoine Poncet, Baris Gencer, Marc Blondon, Marianne Gex-Fabry, Christophe Combescure, Dipen Shah, Peter J. Schwartz, Marie Besson, François R. Girardin

Fig 1 is incorrect in the published article. Please view the correct Fig 1 and its legend here.

**Fig 1:**
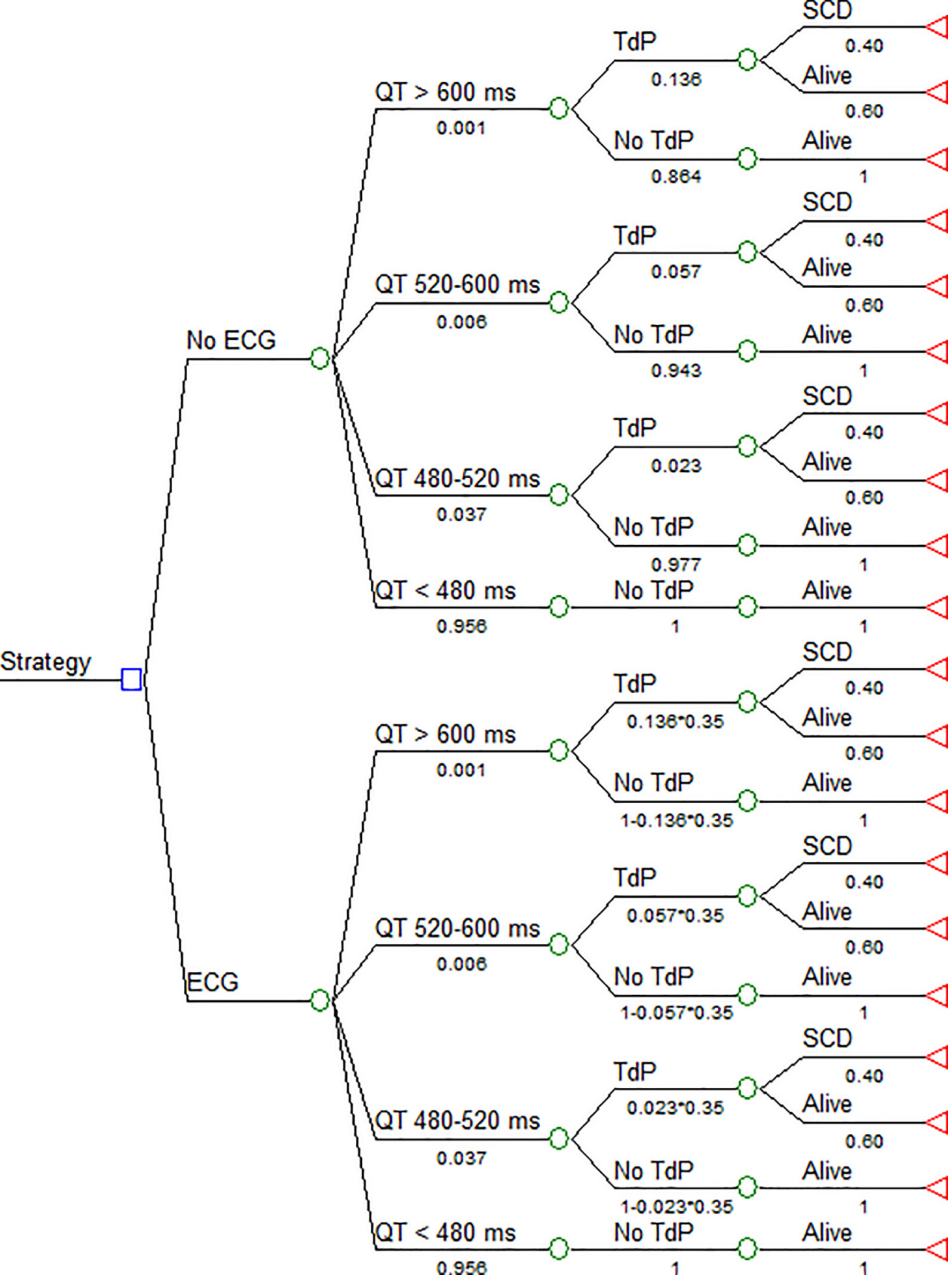
The decision tree represents both strategies: “ECG screening” at hospital admission *versus* “No ECG screening”. Probabilities of patients belonging to a QT category are identical in both strategies, as the risk of SCD after a TdP event. The probability of developing TdP is based on the severity of QT prolongation and is reduced by LQT detection in the ECG strategy. For patients remaining alive, the model assumes a life expectancy of 25 years.
