# Supplementary figures and images for: Electrocardiographic Screening for Prolonged QT Interval to Reduce Sudden Cardiac Death in Psychiatric Patients: A Cost-Effectiveness Analysis
Source: PLoS One. 2015 Jun 12;10(6):e0127213. doi: 10.1371/journal.pone.0127213 (PMC4466505; doi:10.1371/journal.pone.0127213)

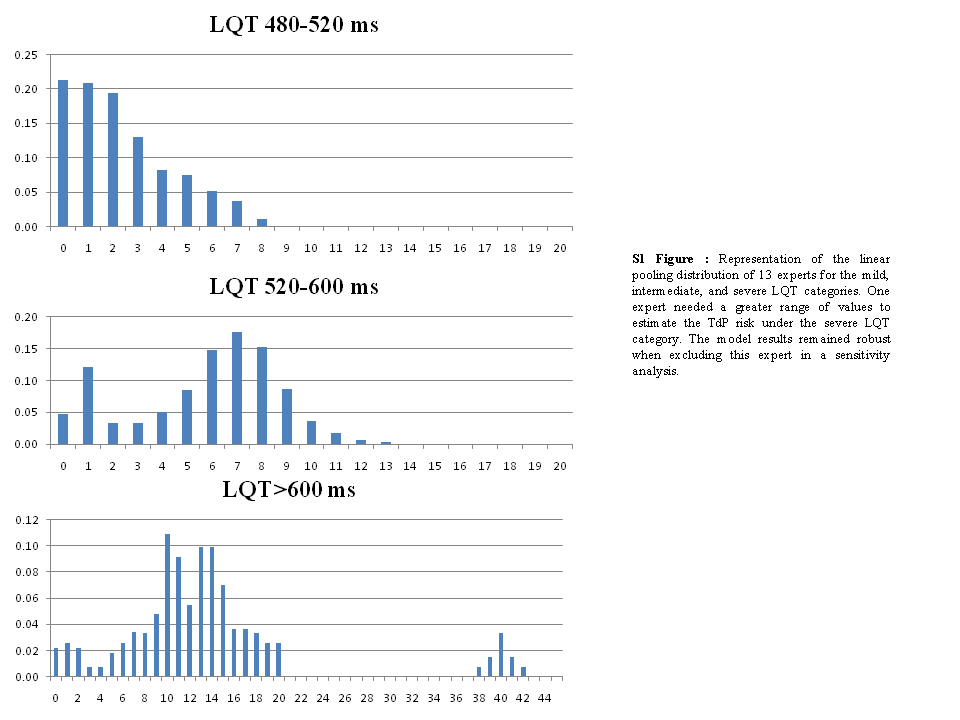

Supplement: S1 Fig — (TIF) [file pone.0127213.s001.tif]

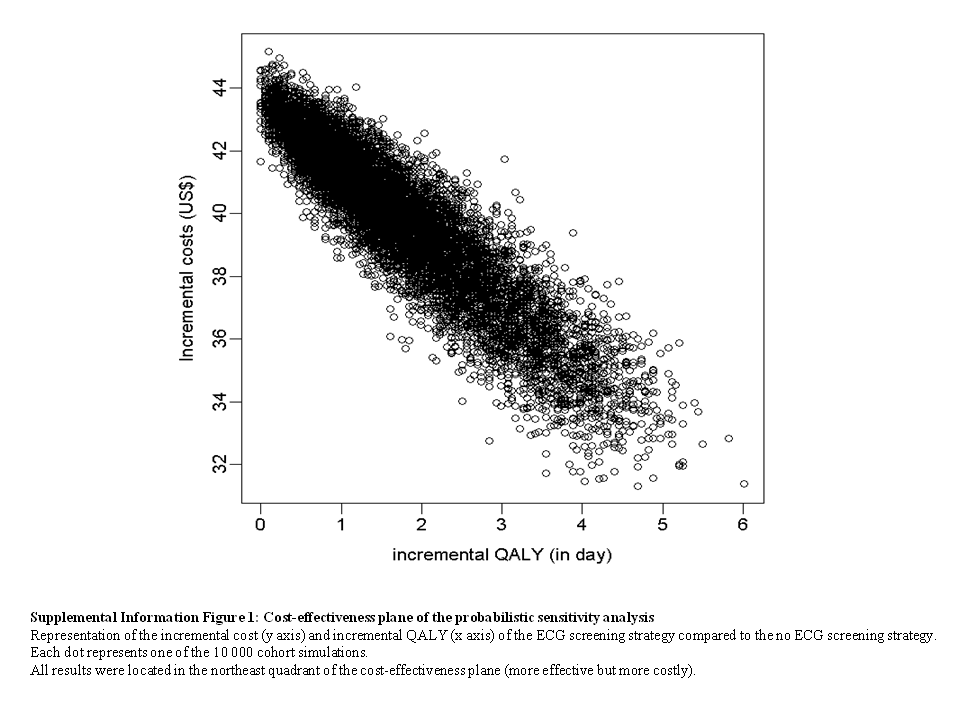

Supplement: S2 Fig — (TIF) [file pone.0127213.s002.tif]
