# Supplementary material for: Electrocardiographic Screening for Prolonged QT Interval to Reduce Sudden Cardiac Death in Psychiatric Patients: A Cost-Effectiveness Analysis
Source: PLoS One. 2015 Jun 12;10(6):e0127213. doi: 10.1371/journal.pone.0127213 (PMC4466505; doi:10.1371/journal.pone.0127213)
Supplement: S1 Table — (PDF) [file pone.0127213.s003.pdf]

Supplemental Information

S1 Table

Experts' elicitation #1

Imagine 100 Patients with a **<u>QTc 480-520 ms</u>** (HR=60/min) on the routine admission ECG (no symptoms)
How many patients will develop TdP during the following year? You can express your certainty or uncertainty by attributing 21 crosses in the figure below.

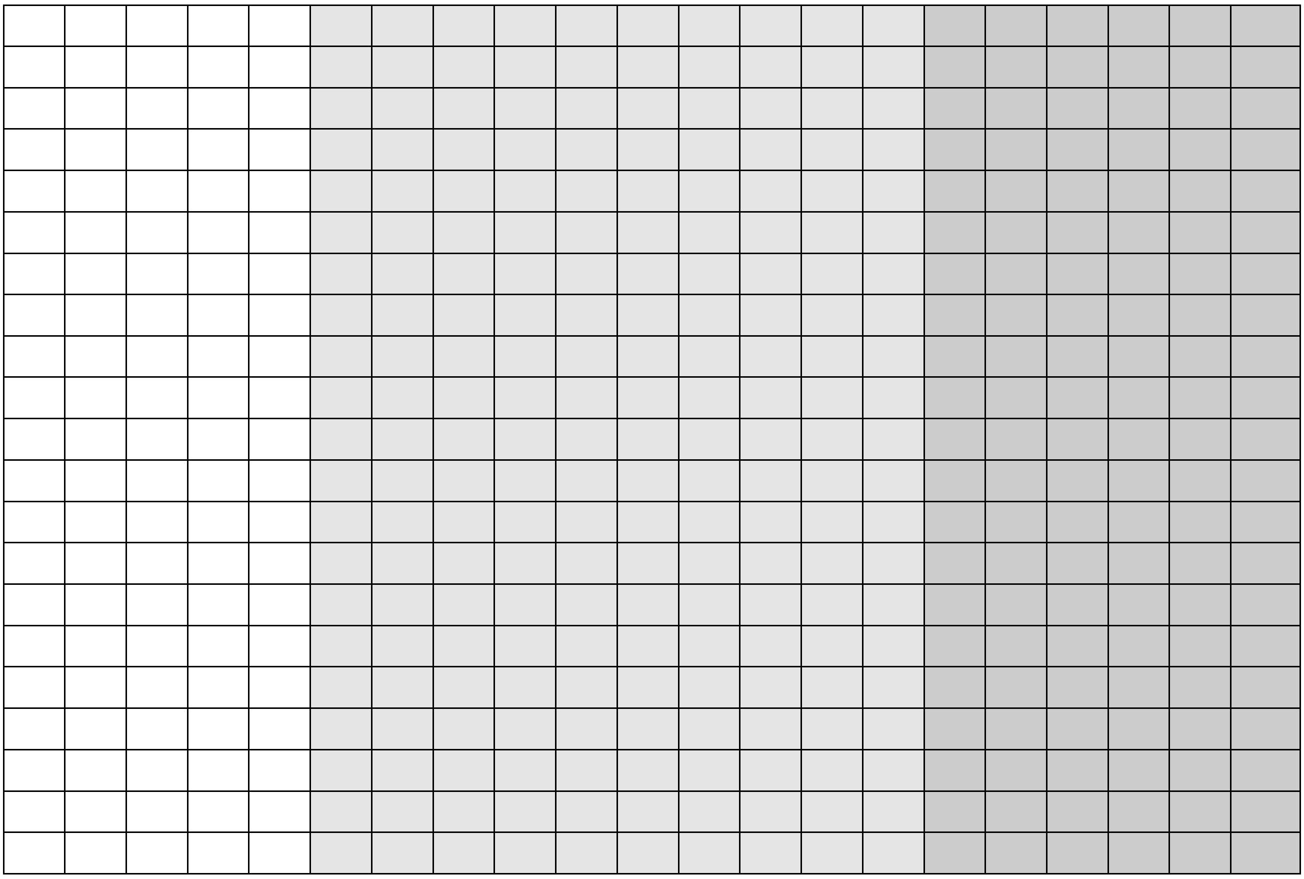

0 1 2 3 4 5 6 7 8 9 10 11 12 13 14 15 16 17 18 19 20

Number of Patients Presenting TdP during the Year Among 100 Patients Hospitalized with a long QTc

Experts' elicitation # 2

Imagine 100 Patients with a <u>**QTc 520-600 ms**</u> (HR=60/min) on the routine admission ECG (no symptoms)
How many patients will develop TdP during the following year? You can express your certainty or uncertainty by attributing 21 crosses in the figure below.

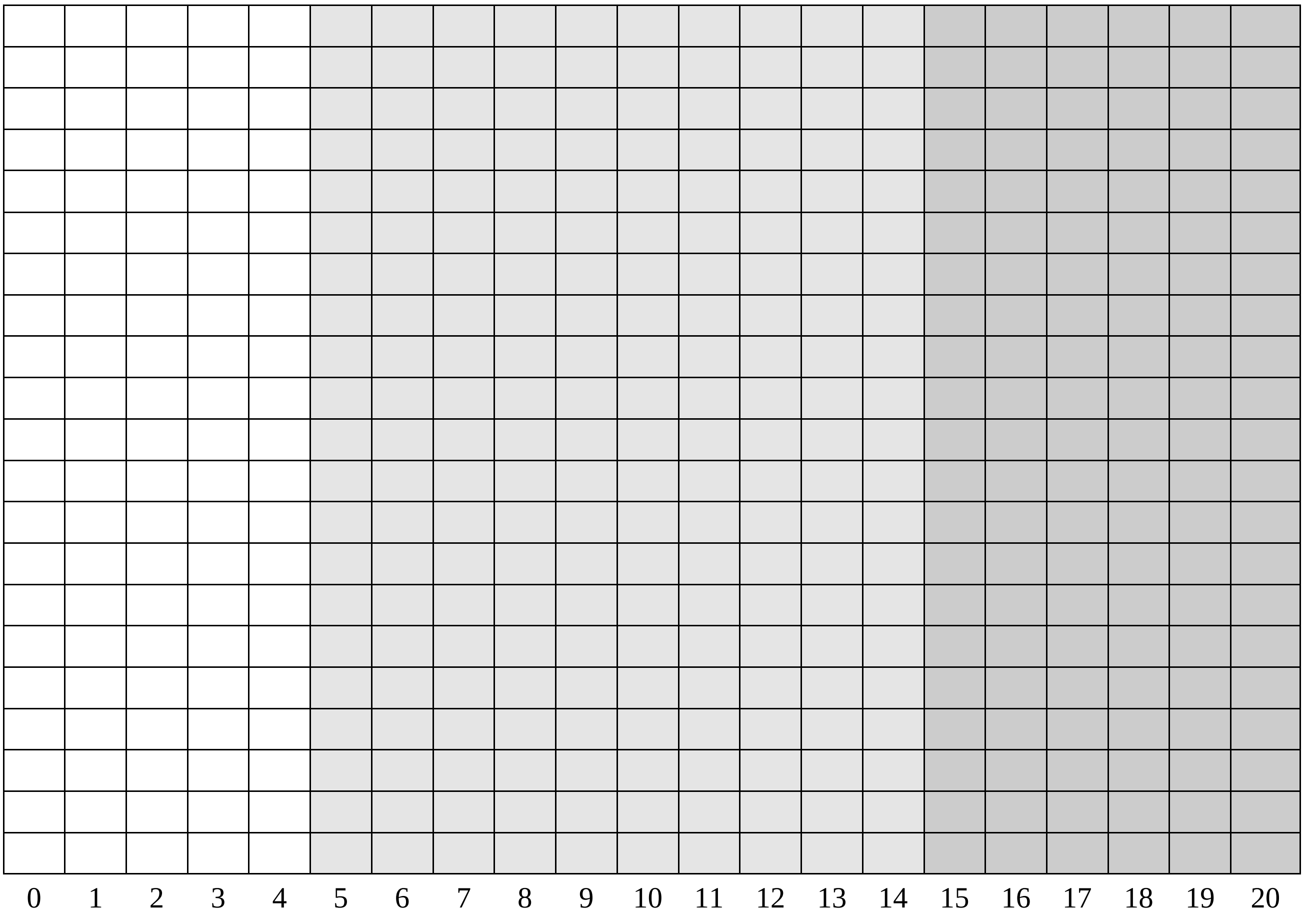

Number of Patients Presenting TdP during the Year Among 100 Patients Hospitalized with a long QTc

Experts' elicitation #3

Imagine 100 Patients with a **<u>QTc > 600 ms</u>** (HR=60/min) on the routine admission ECG (no symptoms)
How many patients will develop TdP during the following year? You can express your certainty or uncertainty by attributing 21 crosses in the figure below.

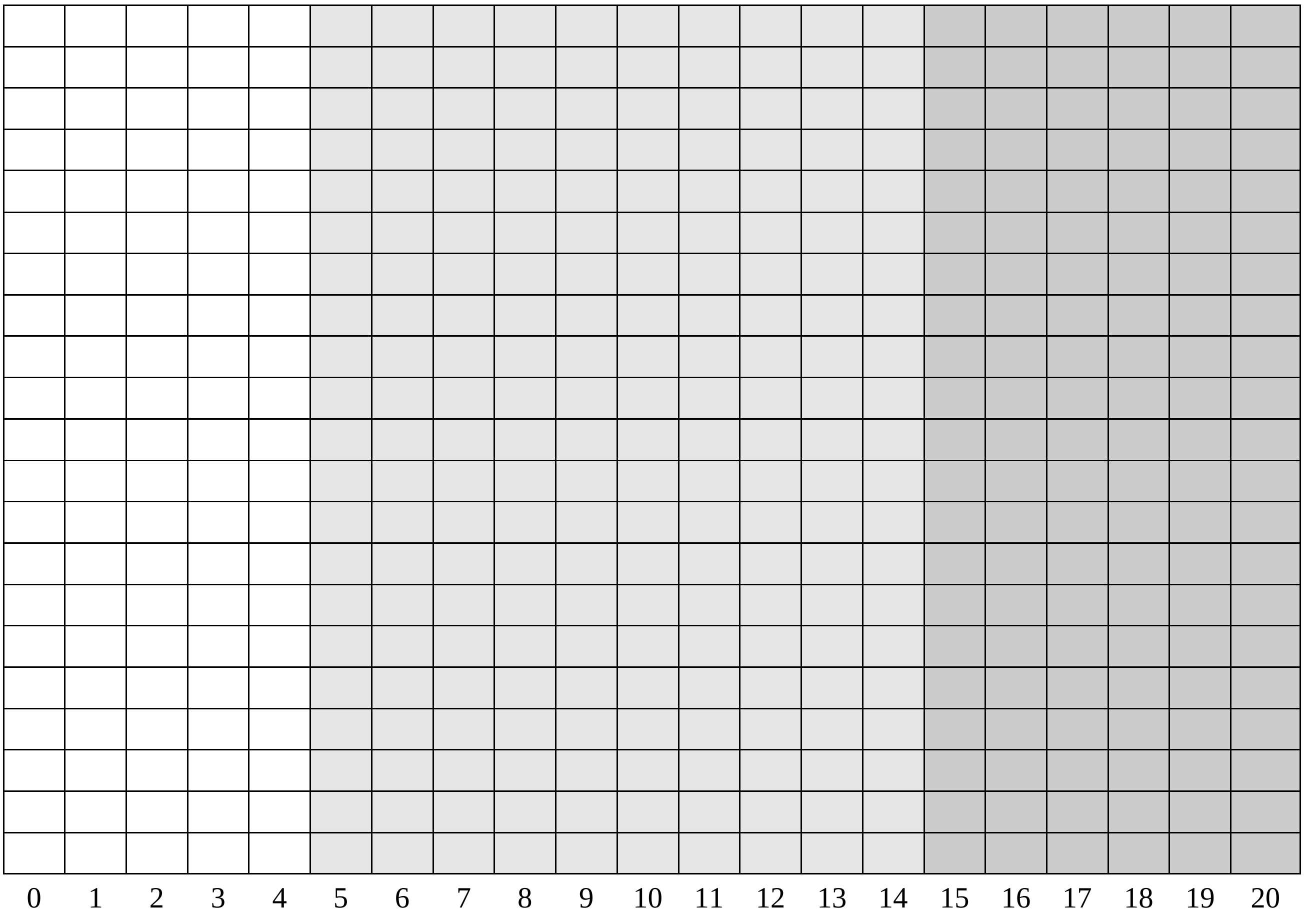

Number of Patients Presenting TdP during the Year Among 100 Patients Hospitalized with a long QTc

The global prevalence of long QT was simulated using a beta distribution *B*(302, 6488) based on the ESOP study reporting 302 patients with a long QT (QT>480ms) among 6 790 patients. Among the patients with a long QT, the proportion of low LQT (QT in 480-520ms) was simulated using a beta distribution with parameters corresponding to 253 observed low QT among 302 patients with LQT. The conditional probability of intermediate LQT was simulated using a beta distribution with parameters corresponding to 42 observed intermediate QT among 49 patients with QT>520ms. The probability of intermediate LQT was obtained by the product of this conditional probability and 1 minus the probability of low LQT. Finally, the probability of high LQT was obtained by complement of low and intermediate LQT probability. With this procedure the sum of probabilities equals 1.
